# Plasmid conjugation drives within–patient plasmid diversity

**DOI:** 10.1099/mgen.0.001361

**Published:** 2025-03-20

**Authors:** Fan Grayson, Leo Loman, Toby Nonnenmacher, Diane Pople, Jack Pollard, Bharat Patel, David Williams, Luke Hounsome, Katie L. Hopkins, Julie V. Robotham, Alice Ledda

**Affiliations:** 1 Advanced Analytics, Analysis & Intelligence Assessment, Chief Data Officer Group, UK Health Security Agency, London, UK; 2 Chief Data Officer Group Private Office, Chief Data Officer Group, UK Health Security Agency, London, UK; 3 HCAI & AMR Modelling and Evaluation, AMR & HCAI Division, UK Health Security Agency, London, UK; 4 Public Health Microbiology Division, Science Group, UK Health Security Agency, London, UK; 5 Antimicrobial Resistance and Healthcare Associated Infections (AMRHAI) Reference Unit, Public Health Microbiology Division, UK Health Security Agency, London, UK; 6 Antimicrobial Resistance and Prescribing, AMR & HCAI Division, UK Health Security Agency, London, UK; 7 National Institute for Health and Care Research (NIHR) Health Protection Research Unit in Healthcare Associate Infections and Antimicrobial Resistance, University of Oxford, Oxford, UK

**Keywords:** antimicrobial resistance (AMR), carbapenem resistance, genomic epidemiology, plasmids, public health

## Abstract

Plasmids are well-known vehicles of antimicrobial resistance (AMR) gene dissemination. Through conjugation, plasmid-encoded AMR genes are spread among neighbouring bacteria, irrespective of their strain or even their species. This process is very concerning from a public health perspective, as plasmid-borne AMR gene outbreaks are often not confined to single species or strains and are therefore more difficult to fully uncover. At the moment, the impact of plasmid conjugation on within–patient plasmid diversity is not well understood. In this work, we will tackle the role of conjugation on within–patient plasmid diversity using a dataset of carbapenemase-producing *Enterobacterales*. The dataset of 256 sequences originates from bacterial isolates cultured from 115 English patients over 30 months. Each patient has more than one sequence, with at least one sequence carrying an OXA-48 gene, a well-known plasmid-borne carbapenemase-encoding gene. If more than one sequence carries the OXA-48 gene, they are carried in different bacterial hosts. Using a hybrid *de novo*–on-reference assembly pipeline, we were able to reconstruct the full OXA-48 plasmid from short read sequencing data for 232 of the 256 sequences. Of the 115 patients, 83 (72%) patients had an identical OXA-48 plasmid in two or more sequences. Only two patients carried very different (*>*200 SNPs) alleles of the OXA-48 plasmid, probably from separate acquisitions. Our study shows that when more than one bacterial host carrying an OXA-48 plasmid is found in a patient, it is most likely that the same plasmid has been shared via conjugation. The event of separate acquisition of different plasmids in different bacterial hosts is highly unlikely in our dataset.

**Impact Statement**

Conjugative plasmids are well-known vessels of horizontal gene transfer, with a prominent role in the spread of antimicrobial resistance genes among different bacterial species or strains. At the epidemiological level, conjugation combined with sequencing a single colony per patient results in plasmid outbreaks carrying antimicrobial resistance genes being found in different bacterial species and strains in different patients, potentially eluding surveillance protocols based on the same bacterium/same resistance scheme. In this study, we analyse within–patient plasmid diversity in a dataset with more than one sequence per patient. Only 2 patients show clear genomic signs of separate plasmid acquisition, while 83 patients share identical plasmids in different bacterial hosts. This points out a very strong role of plasmid conjugation in shaping within–patient plasmid diversity.

## Data Summary

We use data provided by Hopkins *et al.* [1]. The data can be accessed from the National Center for Biotechnology Information and can be found at Bioproject Accession no. PRJNA788733. None of the data used were synthetically generated.

## Introduction

From a public health perspective, plasmid conjugation is a very concerning process. Plasmid conjugation is the process by which plasmids replicate themselves and spread to neighbouring bacteria [2], facilitating the sharing of genetic material between unrelated bacterial cells [3,5]. Conjugation is one of the processes widely known as horizontal gene transfer and can occur across bacterial strains, species or genera [6,9]. Notably, plasmid conjugation enables the dissemination of plasmid-encoded resistance genes among various bacterial species within the same patient [10,13].

This poses a significant challenge in investigating plasmid-mediated antimicrobial resistance (AMR) outbreaks. Typically, AMR outbreaks are characterized by an excess of a specific resistance above a predefined ‘threshold’ value in a specific bacterial host. However, when resistance is plasmid-encoded, it can easily be found in multiple bacterial hosts in the same patient, which may be both different bacterial species or just different bacterial strains. Consequently, sampling a single colony per patient may result in the resistance appearing in different strains in different patients, solely due to the conjugation ability of the plasmid, not as a pre-existing property of the pathogen. In such cases, AMR outbreak characterization based on the single host-single resistance approach may only count sequences where the resistance is found in the specific outbreak bacterium, potentially misclassifying other sequences as non-outbreak-related.

Phylogenetics is a useful tool in tracking outbreaks of diseases and involves analysing the genetic relatedness of outbreak isolates based on their predicted evolutionary relatedness. With epidemiological data, this can provide a better understanding of how outbreaks develop [14] and, in conjunction with comparative genomics, can even help to understand underlying molecular mechanisms and vulnerabilities of the pathogen [7,14].

In the context of plasmid-encoded AMR outbreaks, standard substitution-based phylogenetic analysis of bacterial hosts proves ineffective, as the presence of the plasmid represents an incidental occurrence, rather than the primary evolutionary driver of strains’ divergence. Conducting phylogenetic analysis on the plasmid itself, we must consider that plasmids are very ‘short’ in length and, even considering conjugation, do not replicate often enough during a hospital outbreak to get enough neutral mutations to resolve a full tree [15]. However, plasmids can still accumulate enough adaptive mutations to resolve a full tree to learn about the plasmid’s evolutionary history beyond the simple plasmid typing and resistance identification strategy [15]. Such a tree cannot be used to establish when the plasmid clone originated, since it does not contain time-related information. However, it can still be used to identify groups of related sequences (‘subclades’), which, in connection with epidemiological features, such as location and bacterial host, specific to each subclade, can still provide a better understanding of outbreak dynamics.

Currently, the main problem with the widespread usage of plasmid phylogenetics is plasmid reconstruction [16]. Short read sequencing is not ideal for reconstructing plasmid sequences, as they are circular, often incorporate repeated sequences longer than the read length (thus are difficult to resolve), and it is not easy to account for all events of gene acquisition and loss in the subsequent analysis. Until recently, long read sequencing technology has been insufficiently accurate for useful phylogenetic analysis, although this can be solved using hybrid sequencing. While technology is rapidly improving, in the case of a very well-conserved plasmid such as pOXA-48, we can still attempt plasmid reconstruction from short read sequencing data [15].

Carbapenem resistance genes (e.g. OXA-48-like, KPC, NDM and VIM families) are often encoded on plasmids [14,15, 17]. Carbapenems are ‘antibiotics of last resort’ against multidrug-resistant infections [18]. In recent years, carbapenem-resistant infections have become more common, prompting the World Health Organization to classify carbapenem-resistant Gram-negatives as a critical priority for new antibiotic development [19]. Gaining a better understanding of how plasmids carrying carbapenem resistance genes spread is therefore of paramount importance to devise effective strategies to curb their spread.

While plasmid-encoded AMR genetic determinants are spread by plasmid conjugation into different bacterial hosts, the rate at which conjugation happens at the epidemiological level is still quite unclear. Many experiments have been done to determine which plasmids conjugate more often than others and how often they conjugate, but their results are difficult to extrapolate to the epidemiological context [20,21].

Knowing how often we expect to find the same plasmid-encoded AMR genetic determinant in a different bacterial host in the same patient can be useful to fine-tune public health policies for plasmid-encoded AMR surveillance protocols and outbreak investigation. In particular, assessing whether the practice of sequencing a single bacterial colony per patient can be too restrictive and lead to an underestimation of the outbreak size. For this reason, we analysed a dataset of *Enterobacterales* sequences, chosen to have at least two sequences per patient with at least one carrying an OXA-48 resistance gene. Each sequence is derived from a single bacterial isolate originating from a patient; however, for brevity, we will use the term ‘sequences’. We used this dataset to shed light on how often finding the same plasmid-encoded resistance in a patient is due to within–patient conjugation and how often it is due to a separate acquisition of a bacterium carrying the same resistance gene or even a different one. We reconstructed the pOXA-48 plasmid sequences present in each sequence. By studying the differences in its genetic sequence, we can tell if the plasmid is the same (hence resulting from conjugation) or different (hence resulting from separate acquisition). We use this classification to estimate the extent of conjugation and how often we can expect to find in the same patient plasmid variants so distant that can be explained by separate acquisitions.

## Methods

### Overview

We studied a dataset of within–patient bacterial sequences carrying the OXA-48 gene to better understand the role of conjugation in within-host plasmid diversity. As a general rule, the plasmid can be either vertically inherited, in which case we will find the plasmid in two identical bacterial hosts, same species and same strain (and very little diversity), or it can be horizontally shared by conjugation, in which case we would find the plasmid in two different bacterial hosts that do not necessarily belong to the same species and/or strain. The underlying hypothesis of this work was as follows: if we find an identical plasmid in different bacterial hosts (either species or strain) in a single patient, then the most probable scenario is that the plasmid has been exchanged between the two bacterial hosts through conjugation. If, on the other hand, we find two non-identical variants of the plasmid in two different bacterial hosts, the patient has probably acquired two different bacterial hosts, each independently carrying a variant of the plasmid. We limited our definition of diversity to different bacterial species and/or strains. We built a pipeline to get a reliable plasmid sequence from short read sequencing. The pipeline was developed to minimize the chance of missing small insertions and deletions during assembly. We then aligned the plasmid sequences to a reference sequence (pOXA-48a; JN626286 [22]) and obtained a tree (rooted on the reference plasmid pOXA-48a). We used this plasmid as a reference and an outlier to root the tree, as it was the first plasmid of this type to be isolated and sequenced. It is then fair to assume it is ancestral to our samples and can be used to root the tree. It has also been described as responsible for the international spread of OXA-48 [23] and previously identified in UK isolates [24]. We used the tree to define the different plasmid variants and studied the epidemiological data in the context of these variants and, more generally, the genetic data. We characterized the dataset’s mutation pattern with a focus on the within–patient versus the between–patient pattern. Finally, we used statistical modelling to identify the differences in the distributions of mutations in the within–patient and between–patient pairs of sequences. A full description of the methods is given in File S1, available in the online Supplementary Material. All the scripts used to analyse data and run programs (on a high-performance computing cluster) are available on https://github.com/ukhsa-collaboration/within-patient-pOXA48-conjugation [25].

### Dataset selection, epidemiological data collection and sequencing

Sequence selection and criterion for sequencing are described in full elsewhere [1].

To summarize the selection and criteria, the sequences in this study were derived from bacterial isolates submitted voluntarily by diagnostic laboratories in England to UKHSA’s (formerly Public Health England, PHE) Antimicrobial Resistance and Healthcare Associated Infections Reference Unit between 1 January 2014 and 30 June 2016 for investigation of carbapenem resistance. The first referred bacterial isolate for each patient was sequenced if positive for one or more acquired carbapenemase genes [class A (KPC, FRI, IMI, GES, SME), class B (NDM, VIM, IMP, GIM, SIM, SPM) and class D (OXA-48-like)], as detected by in-house PCR. Subsequent bacterial isolates per patient were sequenced only if they carried a carbapenem resistance gene of a different family or if they carried the same resistance gene but in a different bacterial host. Instances where the same resistance gene was identified in the same bacterial host were not sent for sequencing. We selected all the genomes from patients who had at least one OXA-48-positive bacterial isolate. For each genome, we had access to the original sampling date and site and referring laboratory; the latter were assigned to one of nine geographical areas based on the laboratory location [26].

### pOXA-48 assembly

**Fig. 1.**
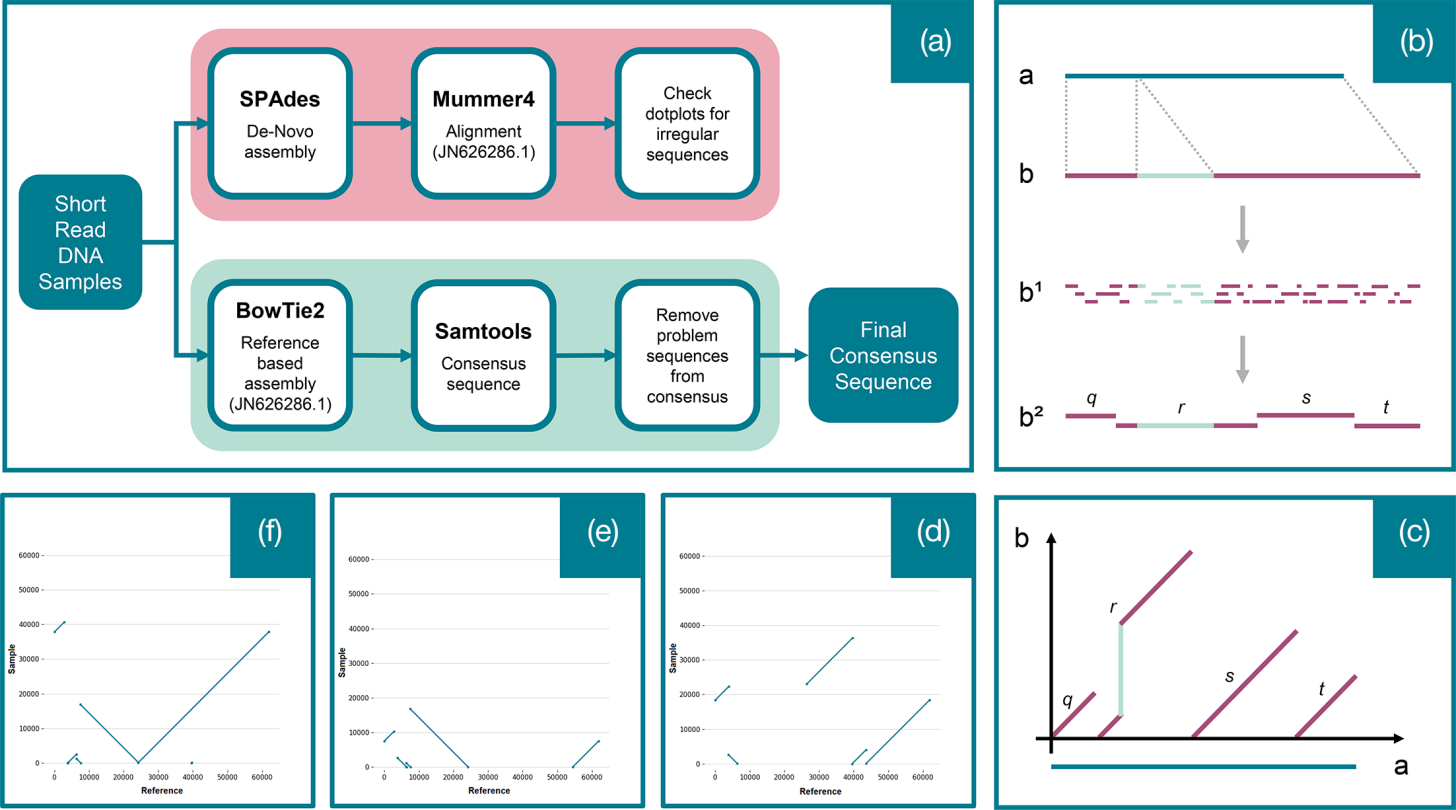
Plasmid reconstruction from short read sequencing. **(a) **Flowchart describing the pipeline used (see File S1 for details). **(b) **Illustration of short read-based *de novo* assembly. Sequence *a* (teal) is the reference plasmid sequence obtained from NCBI (JN626286), and sequence *b* (mauve) is a putative plasmid sequence we need to reconstruct from short read sequences (***b*^1^**). In this case, we assume that plasmid *b* is identical to the reference sequence *a*, except for an insertion (light green). Using *de novo*-based assembly, we construct contigs (***b*^2^**), but we are still not able to circularize the plasmid. The contigs have been labelled *q*, *r*, *s* and *t*. Contig *r* covers the entire insertion (light green), while contigs *q*, *s* and *t* do not have any insertions compared to reference sequence *a*. (**c) **The vignette shows how, for this case, we would be able to identify the insertion by mapping the contigs on to the reference plasmid in a dotplot. The *de novo* assembled contigs (*q*, *r*, *s* and *t*), obtained from vignette B, are mapped to the reference sequence *a* (on the *x*-axis). Contigs *q*, *s* and *t*, each with no insertions, are mapped as unbroken diagonal lines with a slope of 1 (i.e. each base in the contig corresponds to a base in the reference and vice versa) starting at 0 on the *y*-axis (contig) and at the beginning of their location in the reference on the *x*-axis. Contig *r*, which contains an insertion, is also mapped diagonally starting at *y*=0. It is unbroken except in the area of the insertion. The insertion appears as a vertical gap (illustrated in light green), as the insertion region does not map onto the reference sequence and shifts the rest of the contig further up. **(d) **Example of a dotplot for plasmid showing a deletion and probably an inversion. **(e) **Example of a dotplot for a plasmid showing a deletion. **(f) **Example of a dotplot for a plasmid showing a fully reconstructed plasmid (the last contig, starting from ∼24 kb, has been reconstructed in the reverse strand).

A hybrid *de novo*–on-reference pipeline was developed to obtain a reliable sequence of the pOXA-48a plasmid. A schematic drawing of the pipeline is given in Fig. 1(a). The rationale for this is the following: pOXA-48 is usually a very well-conserved plasmid, but it is still subject to a small degree of variability [10]. Consensus sequence among reads mapped to a reference sequence using short sequencing reads could have missed small insertions. The *de novo* pipeline running in conjunction with the on-reference pipeline provides a check for the absence of small insertions included in a contig where part of the plasmid was, as it happens in contig *r* in Fig. 1(b). This made the on-reference assembled plasmid more reliable. It is an approach that works very well for pOXA-48 plasmids, where only small insertions and deletions are present [10]. It is difficult to generalize this method for longer plasmids with bigger insertions and deletions that could get assembled in a separate contig.

#### The *de novo* assembly pipeline

The *de novo* pipeline first ran SPAdes version 3.8.0 [27] on the short reads for each sequence, then aligned the output contigs to the pOXA-48a reference plasmid sequence (GenBank record JN626286, identical to RefSeq record NC 019154 [22]) using mummer4 [28]. Using the contigs assembled with plasmid SPAdes [29] (SPAdes with the ‘–plasmid’ option) does not yield better results than without. This is possibly due to the fact that the copy number of this plasmid is not high enough to make a stark difference in sequencing coverage (that is what the ‘–plasmid’ option uses to better identify plasmids). The *de novo* assembly pipeline using SPAdes without the –plasmid option was not able to reconstruct any or enough contigs for 35 of the 256 sequences, 10 of which were able to be fully assembled by the on-reference method. Using SPAdes without the –plasmid option produced contigs for 218 out of the 232 plasmids that were able to be fully assembled by the on-reference method.

#### The on-reference assembly pipeline

The on-reference assembly pipeline consisted of mapping short reads to the reference sequence with Bowtie2 version 2.2.5 [30,31], then calling the consensus sequence of the mapped reads relative to the reference using samtools – consensus [32].

### Bacterial host identification

Bacterial host identification was done by Kmer-ID (protocol described elsewhere [1]) and confirmed using in-silico MultiLocus Sequence Typing (MLST) using the program MLST [28,33] on whole-genome contigs obtained from SPAdes.

### Alignment and phylogenetic reconstruction

The on-reference assembled sequences were gathered in a single file together with the reference genome and aligned using MAFFT [34]. We developed an algorithm to mask hypervariable regions (see File S1 for details). Briefly, for every position in the alignment, if gaps were found in more than 10% of the sequences at that position, we masked all the mutations in all the sequences in the 50 bp region adjacent to the gap. If gaps were found in less than 10% of the sequences at that position, we masked the mutations in the 50 bp region adjacent to the gap only in the sequences affected by the gap. The rationale for this masking strategy is that if we find a gap in more than 10% of the sequences, we assume that we are dealing with a variable region that may be poorly aligned. However, if we find gaps in less than 10% of the sequences, we assume that the problem is in the genome sequence quality but not in the region.

This masking strategy yielded 247 mutations to resolve the tree.

The masked alignment was used to reconstruct the phylogenetic tree with IQtree version 1.6.12-Linux [35] under the Generalized Time Reversible (GTR) substitution model [36] with the discrete Gamma model [37] with the default four rate categories to correct for rate heterogeneity across sites. The reference plasmid (JN626286 [22]) was used to root the tree, as it is the first pOXA-48 plasmid isolated in 2001, so an ancestor to our dataset.

Figures for all trees were produced using the following R [38] packages: ape [39], ggtree [40], treeio [41] and ggnewscale [42].

### Within–patient diversity

Within–patient diversity was computed using a script in R [38] (see File S1 and github repository [25] for detail).

### Plasmid ancestral state reconstruction

The epidemic clade improved alignment, and the tree was fed to the program augur ancestral [43], which uses maximum likelihood to return the ancestral sequence at each node. Results were analysed and plotted using R [38] (see File S1 and github repository [25]). The reference plasmid was reannotated based on the K8 plasmid in León-Sampedro *et al*. [44], and a genomic comparison between the two plasmids, realised using Easyfig [45] is shown in File S1.

### Comparing within–patient and between–patient diversity

To analyse the distribution of sequences from the same patient in the tree, specifically, the likelihood that each clade contains a pair of sequences from the same patient, statistical tests were performed on the genetic distance between pairs of different plasmid sequences.

The pairwise distances are split into two groups:

**Population 1:** All distances between pairs of different plasmid sequences excluding the ones in Population 2.

**Population 2:** Distances of different plasmid sequence pairs where both sequences in the pair come from the same patient.

The hypothesis test is as follows:

**H_0_:** Population 1 and 2 have the same distribution of genetic distance.

**H_1_:** Population 1 and 2 have different distributions of genetic distance.

We performed two non-parametric hypothesis tests, which were the Mann–Whitney test [46,47] and the Wilcoxon Signed-Rank test [48]. These tests were performed in Python utilizing the scipy package version 1.10.1 [49] (using the stats.mannwhitneyu and stats.mediantest functions). The results of these are given in Table 1(a). A parametric hypothesis test was undertaken by using a negative binomial model [50]. This was performed in R using the mass package version 7.3.57 [51]. We chose this model based on the general distribution of the pairwise distances (heavily skewed left) and a few assumptions (genetic distance has to be a non-negative integer value – this is count data, and we cannot have fewer than 0 SNPs, and there is theoretically no maximum limit for genetic distance). The result of this is given in Table 2(a). These same tests were run on the sequences sampled in the North West to discard the hypothesis that we saw more sequence similarity in this region because of an ongoing outbreak. The results of these are given in Tables1(b)  2(b).

**Table 1.** Results of non-parametric hypothesis tests

Test name	Statistic	*P*-value
**(a) All sequences**		
Mann–Whitney	2568077.5	2.45×10^−17^
Wilcoxon Signed-Rank	40.13	2.38×10^−10^
**(b) Sequences sampled from the North West only**		
Mann–Whitney	678135.0	2.59×10^−9^
Wilcoxon Signed-Rank	38.76	4.78×10^−10^

Results of Ppairwise Ggenetic Ddistance Nnon-Pparametric Hhypothesis Ttests (see Ssection ‘3.5Comparing within–patient and between–patient diversity’).

**Table 2.** Results of parametric hypothesis tests

Variable	Statistic			*z*-value	*P*-value
	Value	L.B.	U.B.		
**(a) All sequences**					
Intercept	3.84	3.81	3.88	226.67	*<*2×10^–16^
Population	−1.59	−2.03	−1.09	−6.64	3.18×10^−11^
Dispersion parameter	0.130	0.127	0.132	−	−
*Akaike information criterion (AIC): 168 098*					
**(b) Sequences sampled from the North West only**					
Intercept	2.15	2.09	2.20	77.17	*<*2×10^–16^
Population	−2.67	−3.23	−1.95	−7.92	2.38×10^−15^
Dispersion parameter	0.117	0.113	0.120	−	−
*Akaike information criterion (AIC): 44 269*					

Results of pairwise genetic distance parametric hypothesis tests using negative binomial regression (see section Comparing within–patient and between–patient diversity). Upper (U.B.) and lower (L.B.) bounds for each statistic produce a 95% confidence interval.

### Estimating at risk bacterial host pairs

To assess the potential bacterial host pairings that may pose a greater risk of plasmid conjugation, two bootstrapping analyses were performed. Specifically, we aimed to estimate the credible range of each of the bacterial pairs occurring within the ‘within–patient group where SNPs equal 0’. This is done by randomly sampling the sub-population with replacement 111 times (this is the number of unique patients). To understand how likely the observed pairings are to occur, we randomly sampled across the entire population where SNPs equal 0, then compared the top 1 to 5 pairs of the sampled population to the observed population, regardless of rank. We performed an exact match of the pairs, then calculated the Jaccard index score [52,53] and reported the mean, 95% confidence interval and median.

## Results

### Dataset description

To study the within–patient variability associated with carriage of OXA-48 genes, we filtered data from a previous study [1] to only include sequences from patients that had at least two sequences with at least one identified as carrying OXA-48. The aim of this study is to understand within-host variability associated with OXA-48 carriage and, in particular, how often we expect to find the same OXA-48 plasmid variant associated with OXA-48 carriage, how often we expect to find a different OXA-48 plasmid variant and how often we expect to find an entirely different carbapenem resistance gene.

**Fig. 2.**
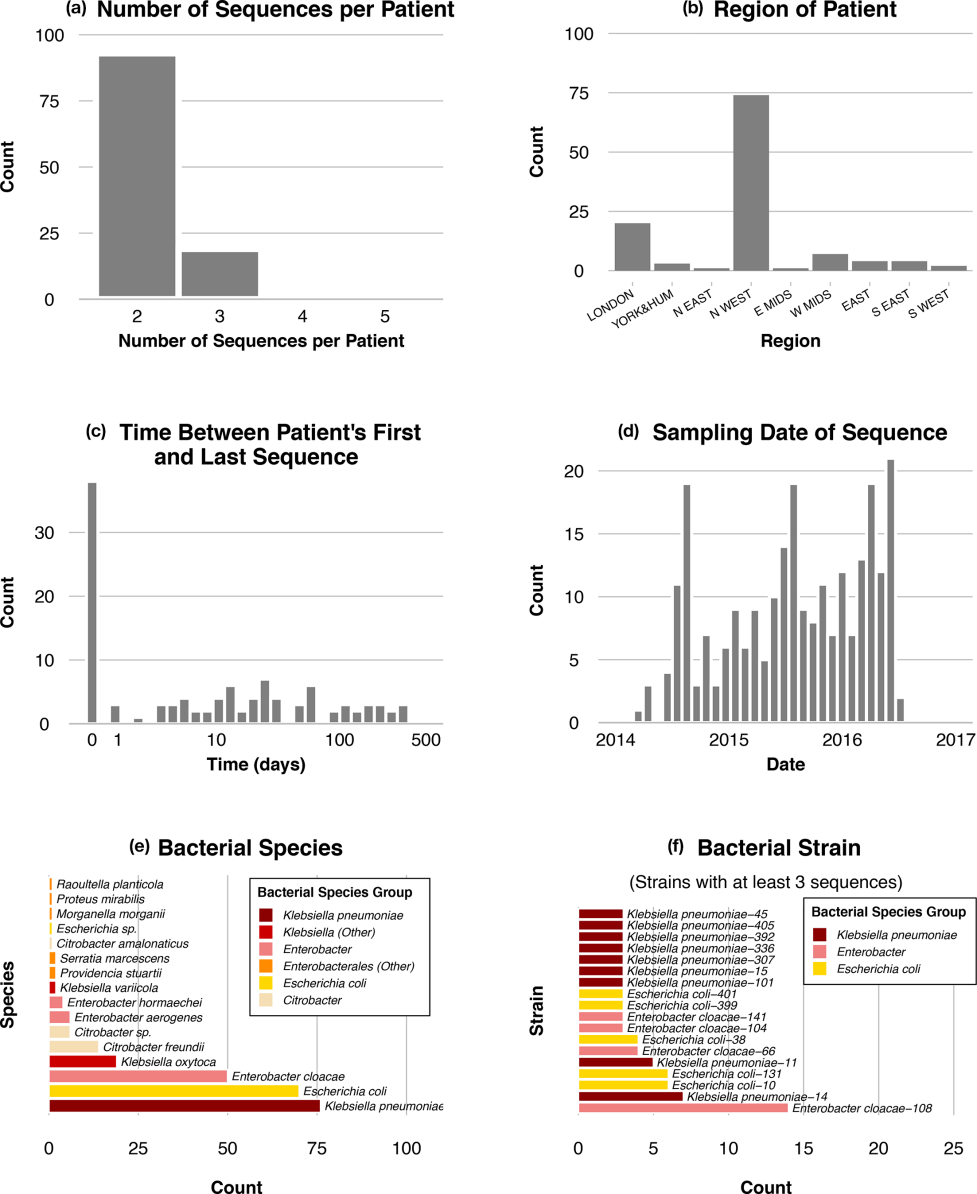
Statistics of the dataset. **(a) **Number of sequences per patient. **(b) **Geographical region of origin of the sequence. **(c) **Within–patient time interval between sequences. **(d) **Date of sampling of each sequence. **(e) **Bacterial species of sequence (coloured by species). **(f) **Bacterial strain (ST) of sequence (coloured by species).

With these requirements, the final dataset consisted of 256 sequences from 115 patients. The median number of sequences per patient was 2 (mean 2.22). The distribution of the number of sequences per patient is shown in Fig. 2(a). The geographical distribution of the referring laboratory location is given in Fig. 2(b) and the sampling dates are shown in Fig. 2(d).

Sequences originating from the same patient were taken at a variable range of time intervals, ranging from the same day up to a 1 year interval (Fig. 2c). A third (38/115; 33.0%) of the patients had all their sequences taken on the same day. The median sampling interval was 9 days (mean 41 days).

In total, 16 *Enterobacterales* species were represented in our dataset (Fig. 2e). The most common were *Klebsiella pneumoniae* (76/256; 29.7%), *Escherichia coli* (70/256; 27.3%), *Enterobacter cloacae* (50/256; 19.5%), *Klebsiella oxytoca* (19/256; 7.4%) and *Citrobacter freundii* (14/256; 5.5%). The complete distribution of bacterial species is given in Fig. 2(e).

Analysis at the strain level provides a more fragmented picture of bacterial host diversity. In the case of a clonal expansion of a specific plasmid-host pair, we would expect a single bacterial strain to be highly represented. However, in our dataset, no strain is highly represented among bacterial hosts, so a clonal expansion of a specific bacterial host does not explain our data. Furthermore, due to the design of the data collection, no patient had multiple sequences of the same strain [1]. Overall, the most common strain found in the sequences is *E. cloacae* ST108 (14/256 sequences; 5.5%), followed by *E. coli* ST10 (6/256; 2.3%), *E. coli* ST131 (6/256; 2.3%) and *K. pneumoniae* ST14 (6/256; 2.3%). All of them are very common strains in their species. The distribution of bacterial strains is given in Fig. 2(d).

Among the 70 *E. coli* sequences present in our dataset, there were 47 sequence types (ST), 36 of which were singletons. The most common *E. coli* strains were ST131 (6/70 sequences; 8.6%), a well-known epidemic and virulent clone, and ST10 (6/70; 8.6%). Other common *E. coli* strains in our dataset include ST38 (4/70; 5.7%), a strain well known to be associated with OXA-48 carriage, albeit often inserted in the chromosome [54], and ST399 (3/70; 4.3%), another strain associated with pOXA-48 carriage in the UK [15]. Seven other *E. coli* STs were represented by fewer than three sequences each. A similar diversity of strains was found for *K. pneumoniae* species. Among the 76 *K*. *pneumoniae* sequences present in the dataset, there were 49 *K*. *pneumoniae* STs, 37 of which were singletons. The most common *K. pneumoniae* STs were ST14 (6/76; 7.9%) and ST11 (5/76; 6.6%). Both strains happen to correspond to the most abundant *K. pneumoniae* strains worldwide. Nine other *K. pneumoniae* STs were represented by two to four sequences each. Among the 50 *E. cloacae* sequences present in the dataset, the most abundant ST was ST108 (14/50; 28%) and accounted for the most common ST in the entire dataset. In total, 19 *E. cloacae* STs were singletons, and 5 other *E. cloacae* STs were represented by two to four sequences each.

### Plasmid reconstruction from short read sequencing

The OXA-48 gene was first described in a 61 881 bp IncL/M-type plasmid, pOXA-48a [22], and tends to be found in IncL/M plasmids of similar length [44] (although it has been described as inserted in the chromosome in *E. coli* ST 38). While other plasmids are well known to be highly variable in their gene content, pOXA-48 is an atypical plasmid in that its gene content is largely well-conserved, and evolution acts mostly in terms of point mutations or by homologous recombination [15]. Still, in large sequence datasets, some degree of variability in pOXA-48 has been observed: DelaFuente *et al*. [10] and León-Sampedro *et al*. [44] in their dataset sampled from 4 hospital wards over 2 years containing over 200 pOXA-48 isolates identified 35 pOXA-48 plasmid variants, mainly characterized by point mutations but also by deletions ranging from a few hundred bps and up to several kbs with their consensus plasmid being 65 499 bps long, ∼3700 bps longer than the plasmid used as a reference in this article [10].

For plasmid reconstruction, an empirical hybrid *de novo*–on-reference pipeline was developed that has been proposed elsewhere [15]. This pipeline is illustrated in Fig. 1(a). The rationale for this hybrid *de novo*–on-reference pipeline is that we expect the plasmid’s genetic content to be well-conserved. More precisely, given the specific conservation features of this plasmid, we expect almost complete synteny except for occasional small (*<*1 kb) insertions. A standard reference-based reconstruction might miss insertions in the plasmid sequence (Fig. 1b). To control for small insertions, we run a *de novo* assembler (Fig. 1b) and then we map its contigs on the reference plasmid (Fig. 1c). Considering that the whole plasmid was often covered by only a few contigs, small insertions would have been visible, mapping the contigs on the reference plasmid in the dotplots as discontinuities in the contig (*y*-axis, Fig. 1c), while deletions would be clearly seen on the dotplot as discontinuities on the reference (*x*-axis, Fig. 1d, e). In Fig. 2(f), we show for comparison an example of a dotplot with no discontinuities (perfect assembly). In this case, the contig running from ∼8 to ∼25 kb is mapped on the reverse strand, hence the downward slope. All the dotplots were visually inspected, and no sign of insertions was detected.

Of the 243 sequences in the dataset identified as encoding the OXA-48 gene, the full pOXA-48 sequence can be reconstructed for 232 sequences (95%) using this hybrid pipeline. Additionally, 6 out of the 243 sequences were identified as encoding both NDM-1 and OXA-48 genes, and 16 sequences were identified as encoding other carbapenemase genes (see Table 3).

**Table 3.** Number of sequences with carbapenem resistances (excluding OXA-48)

Resistance	No. of sequences
NDM-1	5
KPC-2	5
NDM-4	1
OXA-181	1
OXA-244	1
VIM-4	1

By manually reviewing the dotplots, we found three sequences with a contig mapping on the region of the reference plasmid where the OXA-48 gene is, but no other contigs mapping on the rest of the reference plasmid. Of these three sequences, one (PAT09-S020) carried an OXA-181 gene, whose sequence differs from the OXA-48 sequence by only four mutations, therefore mapping on the OXA-48. OXA-181 is known to be carried on a variety of non-IncL/M-type plasmids [17]. The other two sequences carried OXA-48 genes, the first from an *E. coli* ST38 (PAT233-S505), and the second sequence carried OXA-48 in a *Citrobacter* (PAT92-S204). *E. coli* ST38 has been previously described with part of pOXA-48 inserted in its chromosome [55]. Insertion of OXA-48 in a *Citrobacter* chromosome or in a non-IncL/M-type plasmid is also described [56]. These provided a useful check that our pipeline worked properly.

As a consistency check, we ran PlasmidFinder [57] on the whole dataset, as it is the usual standard for plasmid identification. Resulting .json files were parsed using R [38]. PlasmidFinder identified an IncL/IncM plasmid in 221 of the 256 sequences in the dataset. PlasmidFinder results are given in File S1.

**Fig. 3.**
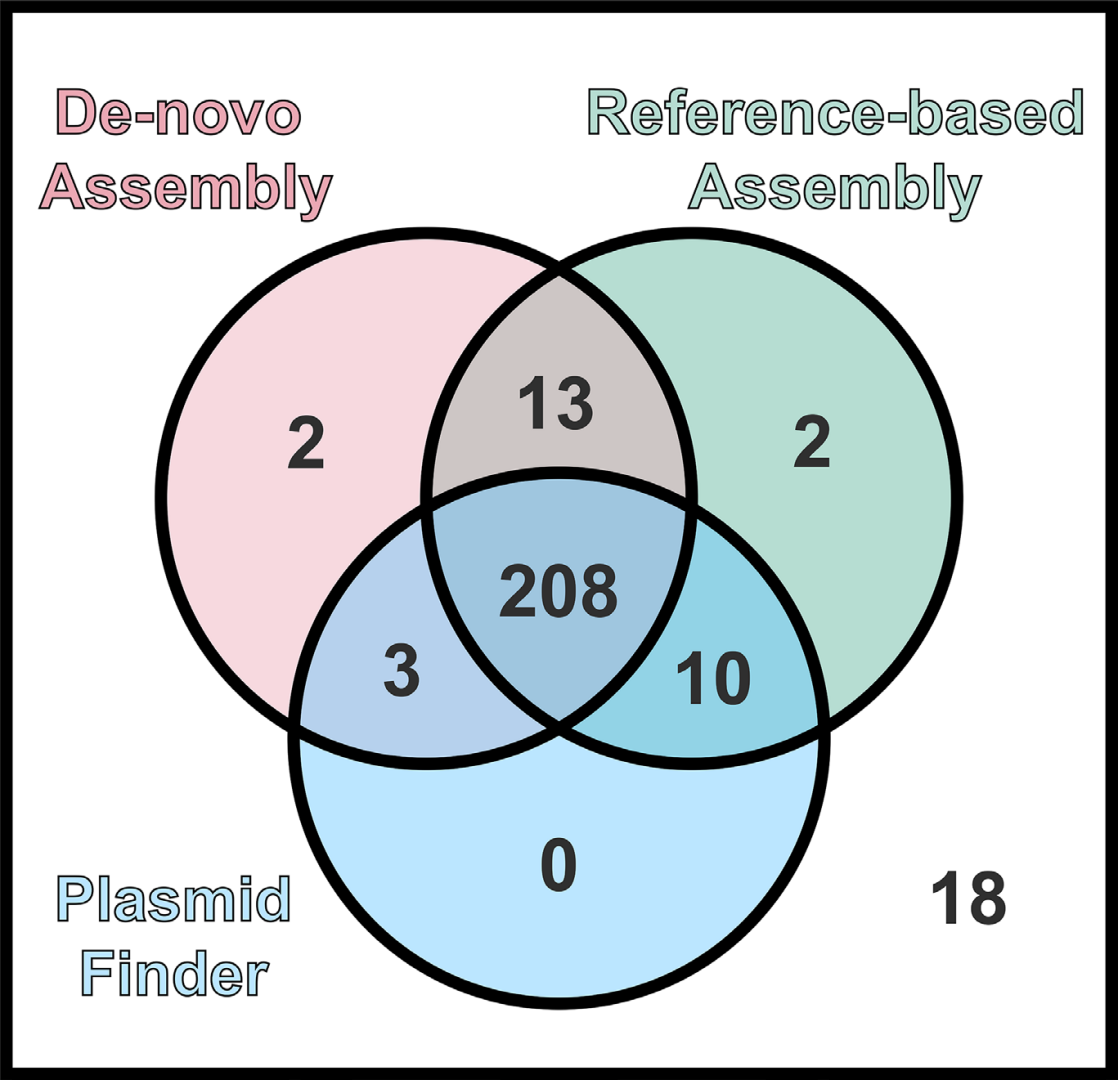
pOXA-48 identification Venn diagram. Venn diagram showing the number of sequences where pOXA-48 is found by each of the different pipelines. The Venn diagram has been colour coded in a similar fashion to Fig. 1(a), with the *de novo* pipeline coloured red and the on-reference pipeline coloured green.

A visual comparison between PlasmidFinder and each part of our hybrid pipeline findings is shown in Fig. 3. The on-reference assembly pipeline was able to fully assemble 232 plasmids out of our 256 sequences. The *de novo* assembly pipeline using plasmid SPAdes was not able to reconstruct any or enough contigs for 30 of the 256 sequences – 10 of these 30 plasmid sequences were able to be fully assembled by the on-reference method. PlasmidFinder identified a pOXA-48 plasmid in 221 out of the 256 sequences. This is compared to 226 sequences found with pOXA-48 using the *de novo* pipeline and 232 using the reference-based pipeline. The *de novo* pipeline identified the pOXA-48 plasmid in two sequences where no other pipeline identified it. The reference-based pipeline identified the pOXA-48 plasmid in two sequences where no other pipeline identified it. PlasmidFinder did not exclusively identify any plasmid. In total, 13 sequences were found by the *de novo* and the reference-based pipeline but not by PlasmidFinder. Three sequences were found by the *de novo* pipeline and PlasmidFinder but not by the reference-based pipeline. Two sequences were found by the reference-based pipeline and PlasmidFinder but not by the *de novo* pipeline. For 208 sequences out of 256, the pOXA-48 plasmid was identified using all three pipelines. All three pipelines agreed that for 18 sequences out of 256, no pOXA-48 plasmid is present.

Having made sure that our pipeline was accurate enough and that no insertions were present in our dataset, all of the plasmid sequences obtained from the on-reference mapping of the reads were used to build the alignment (232/256 sequences).

### Tree analysis

**Fig. 4.**
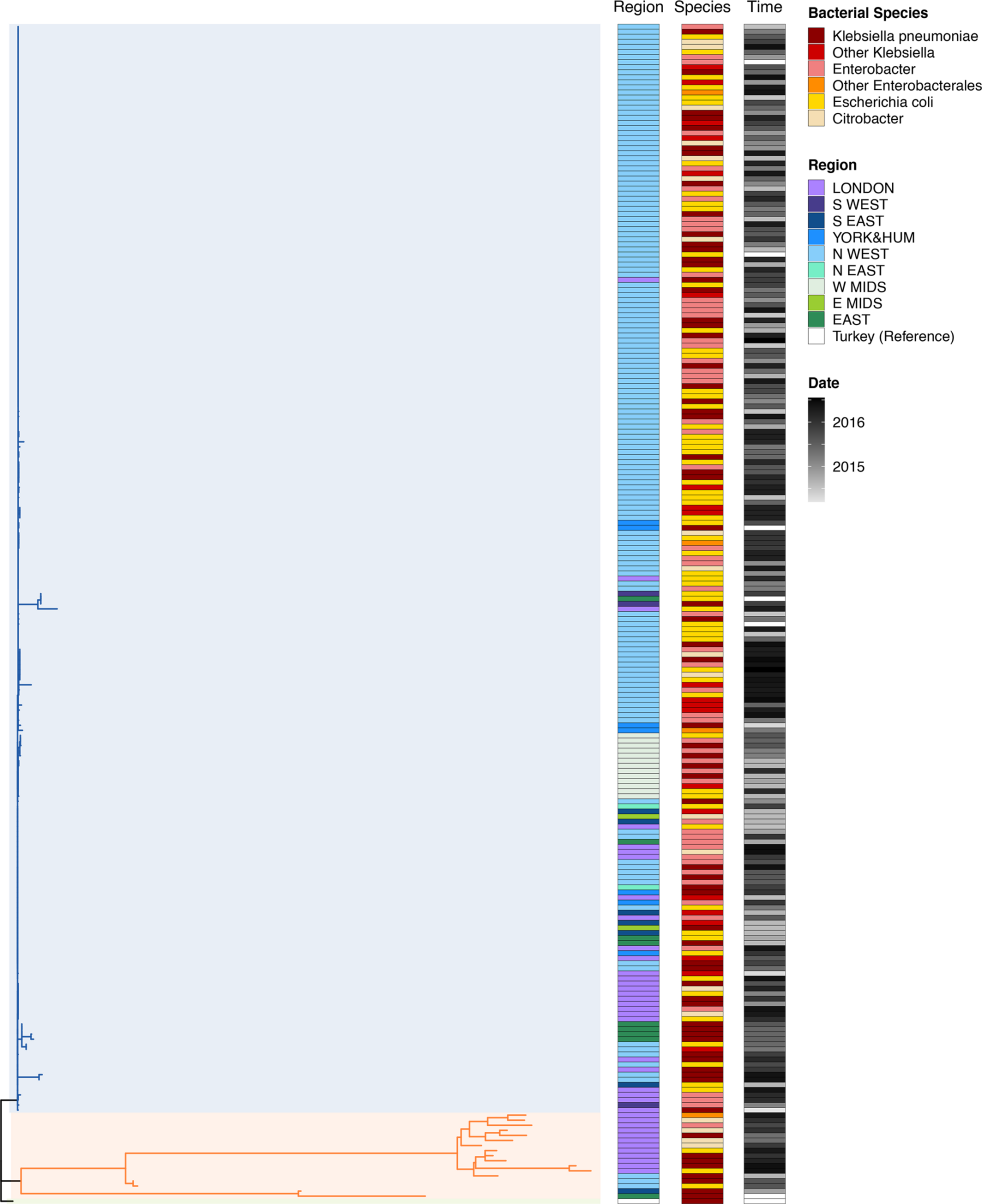
Phylogenetic tree. All pOXA-48 sequences. Phylogenetic tree and analysis of the pOXA-48 sequences. The phylogenetic tree is rooted at the reference plasmid JN626286. It is split at its base into two main clades, shaded in light orange (the ‘diverse’ clade) and light blue (the ‘epidemic’ clade) for easier identification. The three colour-coded columns to the right of the tree show the epidemiological data for each sequence. The first column labelled ‘Region’ (shades of blue, purple and green) indicates the region where the sequence was collected. The second column labelled ‘Species’ (shades of orange, red and yellow) indicates the bacterial host species. The third column labelled ‘Time’ (shades of grey) indicates the sampling time.

To better understand the variability of the pOXA-48 plasmid, we inferred a phylogenetic tree from all 232 of the plasmid sequences we were able to reconstruct using the reference-based pipeline and used the pOXA-48a reference plasmid (JN626286) as an outlier to root the tree (Fig. 4).

Throughout this article, we will define a clade in a tree as sets of closely related sequences originating from a common ancestor. The tree is clearly split into clades, one containing 17 very genetically diverse sequences and another containing 215 very closely related sequences. The former, mainly representing plasmid sequences from bacterial isolates originating in London, may be considered as a sampling of the available pOXA-48 diversity worldwide, while the latter clade is clearly driven by the epidemic pOXA-48 clade expanding in some regions of the UK. From here on we will refer to the first clade as the ‘diverse’ clade (shaded in light orange in Fig. 4) and to the latter as the ‘epidemic’ clade (shaded in light blue in Fig. 4). These clades have different geographical origins: the diverse clade has mostly been sampled in London (*P*-value<0.01, Fisher exact test), while the epidemic clade has mostly been sampled in the North West of England (*P*-value<0.001, Fisher exact test). Sampling time and bacterial host are not as significantly different between the clades (bacterial host: *P*-value=0.012, Fisher exact test; sampling time: *P*-value=0.2, Kolmogorov–Smirnov test).

**Fig. 5.**
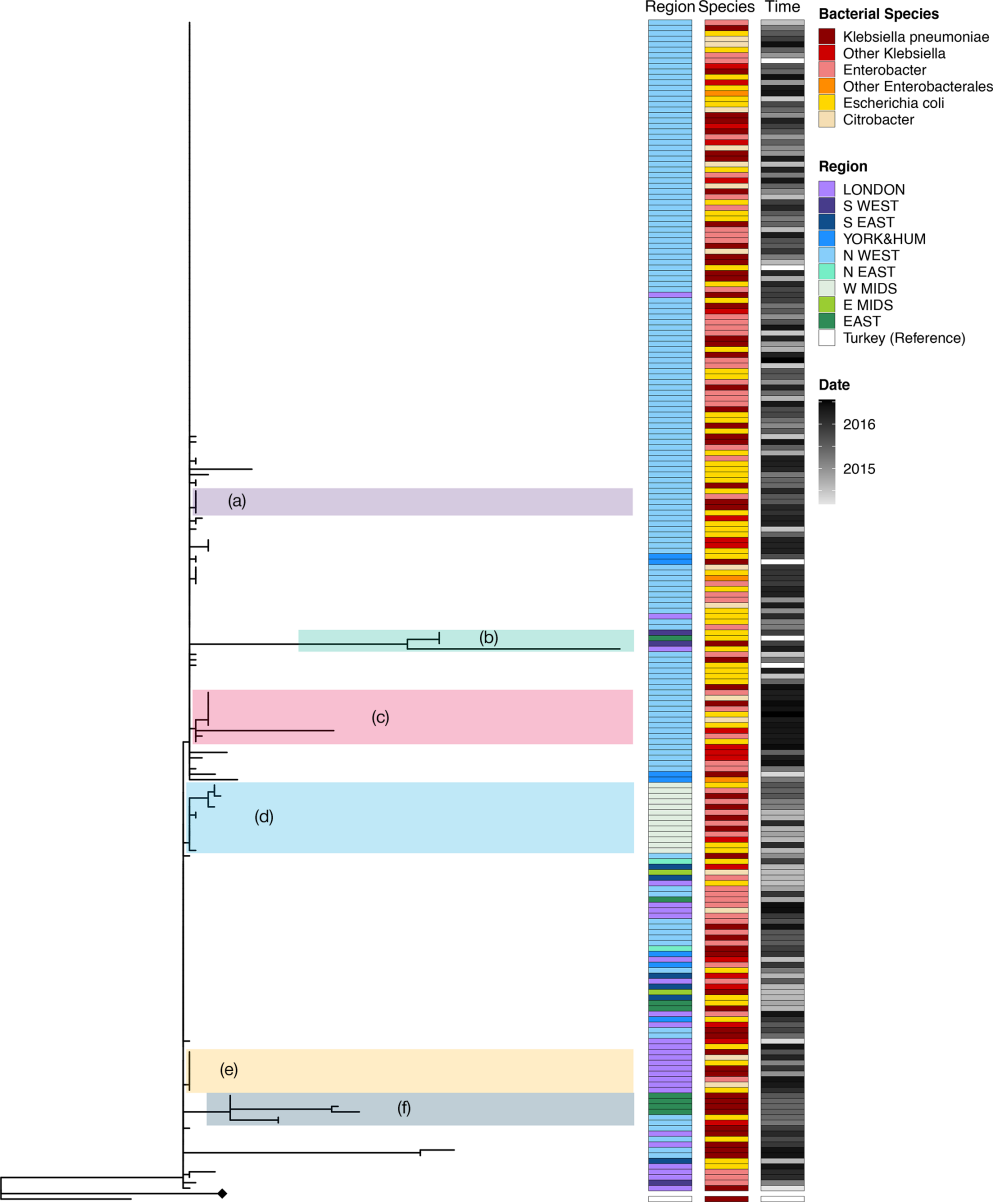
Phylogenetic tree. Subclades within epidemic clade. Detail of the epidemic clade in the tree (a portion of the tree has been collapsed and represented with a diamond node). Columns have the same colour coding as in Fig. 4. The most populated clades are highlighted in different coloured shades and labelled A–F.

A closer look at the epidemic clade (Fig. 5) gives a clearer view of the diversity in this clade. Within the epidemic clade, there is a cluster of 150 identical sequences, while the remaining 65 sequences in this clade display some diversity.

Within this epidemic clade, there are six main subclades (labelled A to F), containing 4 to 13 sequences, and with sequences belonging to more than one patient. While these clades do not show clustering in terms of host species (non-significant *P*-value for each clade, Fisher exact test) or sampling time (non-significant *P*-value for each clade, Kolmogorov–Smirnov test), four of the six clades (B, D, E and F) have a well-defined geographical origin. Clade B has a mixed geographical profile (two sequences from the South West region, one from the East of England and one from London), but still significantly different from the overall geographic profile of the epidemic clade (*P*-value=4×10^−4^, Fisher exact test), clade D is from the West Midlands (*P*-value<10^−10^, Fisher exact test), clade E is from London (*P*-value=10^−5^, Fisher exact test) and clade F, which has a combination of sequences from the North West and the East of England, is still different from the whole epidemic clade (*P*-value=0.0014, Fisher exact test). Clades A and C instead come from the North West, like most of the sequences in the epidemic clade; therefore, the *P*-value from the Fisher exact test is non-significant.

Throughout the dataset, the only epidemiological feature linked to the genetic difference is the geographical origin of each sequence. This points to small outbreaks of resistance spreading in geographically limited regions, but further information would be needed to support this hypothesis.

### Within–patient diversity

A footprint of within–patient conjugation is the presence of identical plasmids in different bacterial hosts within the same patient. To assess the similarity between plasmids within– patients, we computed the number of single nucleotide variants found between any pair of pOXA-48 sequences from the same patient (within–patient pairwise genetic distance). In general, when comparing variability between plasmids, we call each genetic variant a ‘plasmid variant’. When a patient has two identical plasmids (pairwise genetic distance=0), they carry the same plasmid variant. When the patient carries two plasmids with pairwise genetic distance>0, we say that they carry different variants of the plasmid. Two different variants of the plasmid might be evolutionarily related to each other, meaning that they share some of the same mutations, which makes us think that one is an ancestor and the other is a descendant. On the other hand, when two different plasmid variants do not share any mutation, it is unlikely that they are evolutionarily related to each other.

**Fig. 6.**
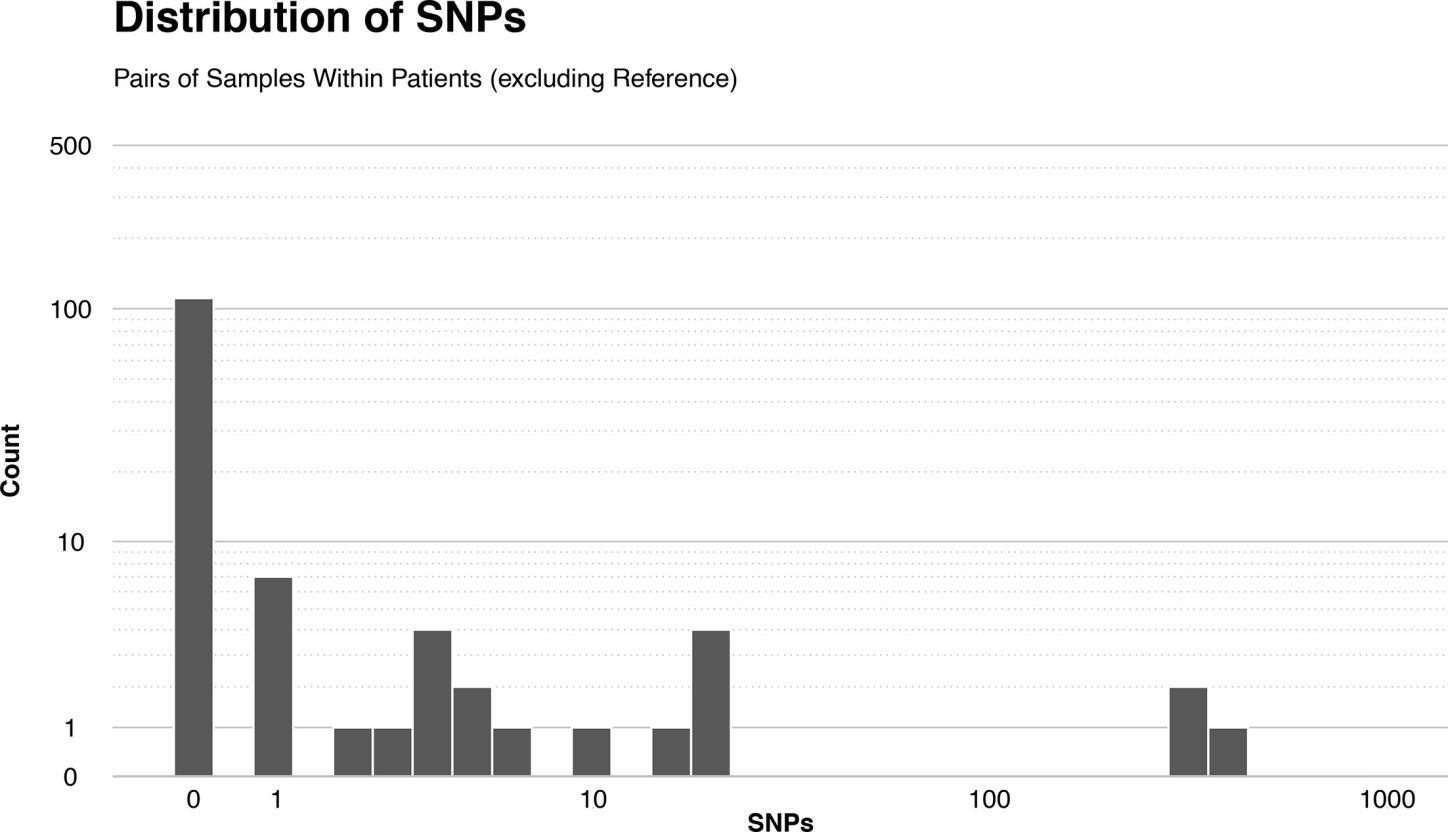
Histogram of within–patient genomic distances.

Given the within–patient distribution of the sequences (see Fig. 1a), we were able to compute 136 within–patient pairwise genetic distances. The distribution of these distances is given in Fig. 6. As expected from the tree in Fig. 4, the vast majority, 111 of 136 (82%), of the sampled patients yielded identical plasmid sequences (genetic distance=0). Of the 136 total sampled patients, 16 (12%) plasmid sequences differ by a handful of mutations (1–6 SNPs). A further six pairwise genetic distances are in the tens of SNPs and a further three distances are in the hundreds of SNPs. The average within–patient pairwise genetic distance is 9.5 SNPs; the median is 0 SNPs.

When looking at the within–patient diversity, we find the same OXA-48 plasmid variant in two or more sequences in 85 of the 115 patients in our dataset (74%). In 83 of these 85 patients (72% of the total), all sequences contained the same variant, indicating no pOXA-48 diversity. In two patients, PAT157 and PAT219, from which three OXA-48-positive bacterial isolates were each isolated, we found two identical variants and one different variant pOXA-48 plasmid. In total, 18 of the 115 (16%) patients carry at least two different plasmid variants. There is no significant difference in sampling time interval in the carriage of identical plasmid alleles or different plasmid variants (Kolmogorov–Smirnov test).

**Fig. 7.**
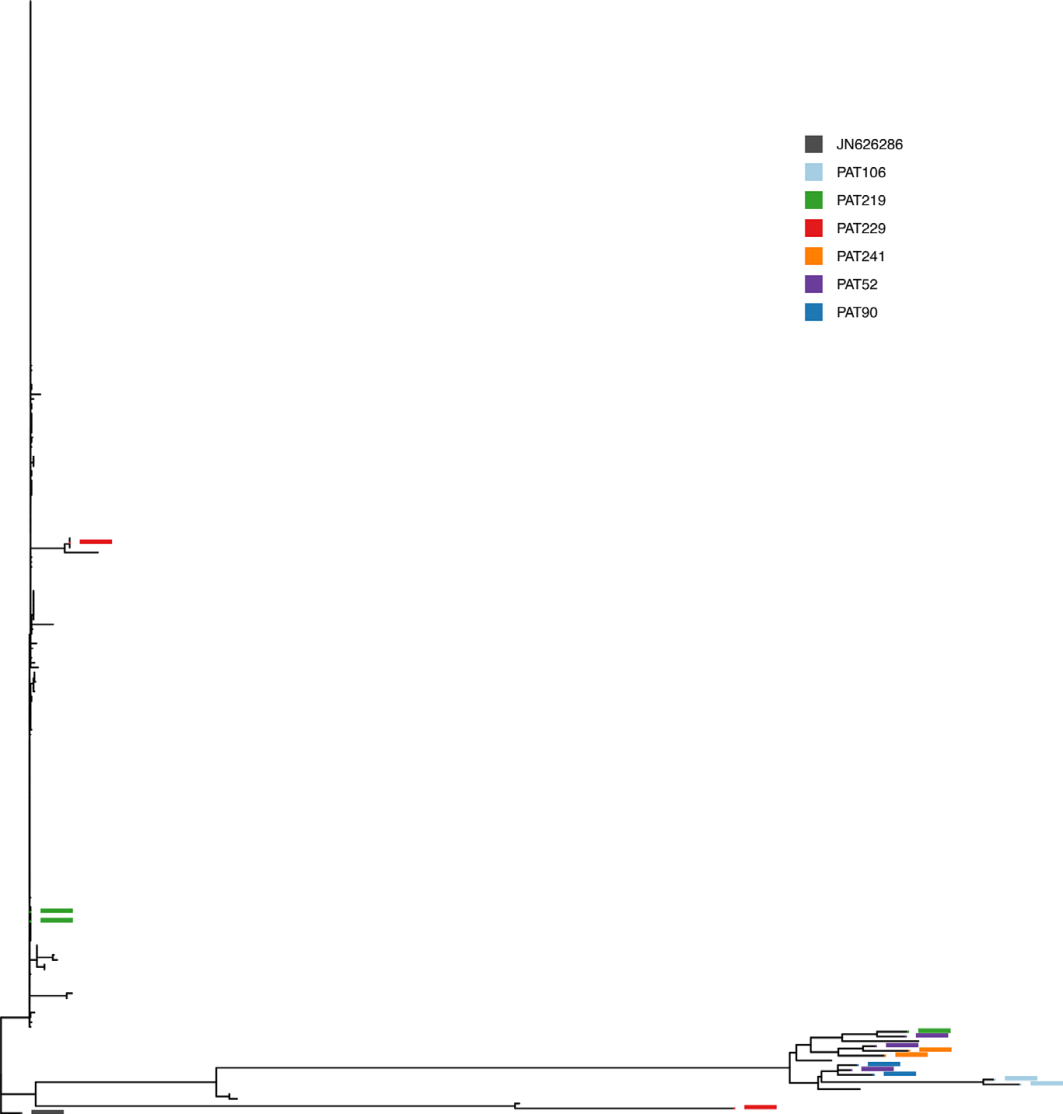
Phylogenetic tree. Patients with sequences containing distinct alleles of the pOXA-48 plasmid, and with at least one sequence in the non-epidemic ‘diverse’ clade. These are good candidates for having acquired the different plasmids on different occasions. Node highlight colours correspond to each patient.

Only two patients have sequences from both the ‘epidemic’ and the ‘diverse’ clade (PAT229 and PAT219, as seen in Fig. 7). Since the two clades are so distinct, this can only happen if the plasmids have been separately acquired. In particular, PAT219 is a textbook example of separate acquisition, as both the different alleles we find in each sequence have another identical plasmid found elsewhere on the tree in sequences from other patients and different bacterial hosts.

**Fig. 8.**
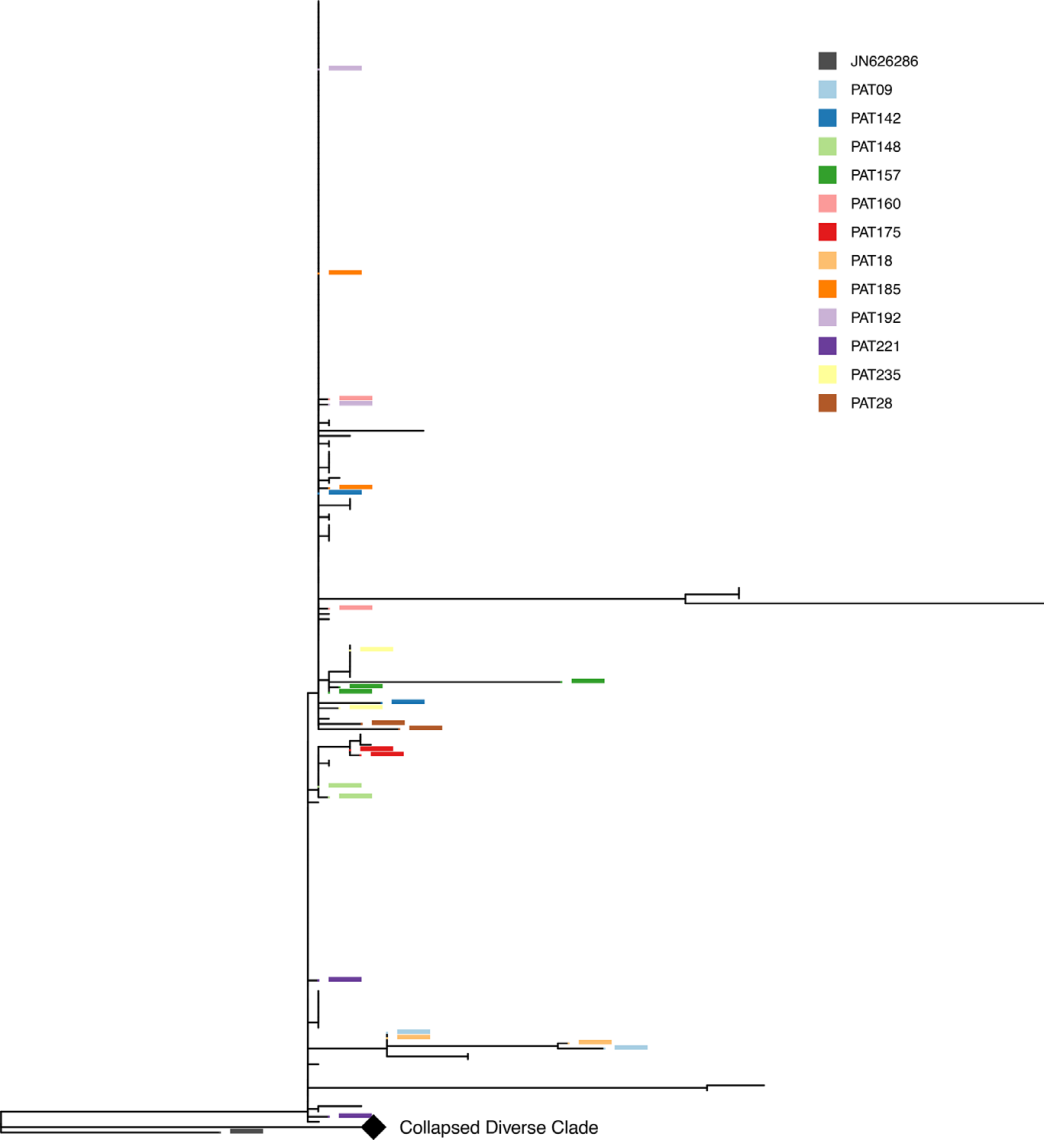
Phylogenetic tree. Patients with sequences containing distinct alleles of the pOXA-48 plasmid, and with *no sequences* in the non-epidemic ‘diverse’ clade. These are good candidates for having acquired the different plasmids on a different occasion. Node highlight colours correspond to each patient. The non-epidemic ‘diverse’ clade has collapsed and is represented with a diamond node.

The rest of the sequences show some diversity, and when plotted on the tree, do not show clear footprints of separate acquisitions. In the case of within–patient evolution, we expect one of the sequences to sit at the base of the clade and the other at the tip. Examples of such dynamics are PAT157, PAT09 and PAT18 in Fig. 8. For other sequences, like those from PAT106, PAT241 and PAT90 in Fig. 7, it is clear that the sequences are somehow related through their evolution, but more data would be required to determine whether this is due to within–patient evolution or due to a wide transmission bottleneck from an evolving source.

### Comparing within–patient and between–patient diversity

If the plasmids found within the same patient have been acquired separately from a common set of plasmids (in this case our full dataset), we would expect this to reflect in the distribution of their length. Namely, we would expect the distribution of within–patient plasmid lengths to be non-statistically significantly different from the distribution of plasmid length in the entire dataset. If, on the other hand, conjugation has a role in plasmid spread, plasmids found within–patient would not be randomly sampled from the entire dataset. We would therefore expect the within–patient distribution of plasmid lengths to be statistically significantly different from the global distribution of plasmid lengths in the dataset. The results of the tests can be found in Table 1(b). These tests all show that there is a statistically significant difference between the within–patient plasmid subset and the whole dataset. The tests all indicate that we can reject the null hypothesis, which is that the distributions of the two sets are the same, suggesting that conjugation does have a role in plasmid spread.

To exclude that the increased similarity in within-host plasmids in the epidemic clade was due to sampling from an ongoing outbreak in that region, the same tests have been performed on sequences sampled from the North West only. In an ongoing outbreak, we would expect a recent clonal expansion of the outbreak plasmid clone. Being recent, they would not have had time to acquire mutations (random or adaptive alike); therefore, we expect outbreak sequences to be more similar than non-outbreak sequences. However, *P*-values were still significant, meaning that, despite being sampled in an outbreak, the within–patient similarity was different from the between–patient similarity (see Table 2b). This result points again to the role of plasmid conjugation in within–patient similarity (as opposed to random sampling from the pool of plasmids available).

### Bacterial host pair analysis

Using bootstrapping (see Estimating at risk bacterial host pairs), the mean, median and 95% credible range that may be observed within a wider population for the top five most occurring bacterial host pairs in the ‘within–patient group where SNPs equal 0’ are given in Table 4. We find the top three most occurring bacterial pairs exhibit consistency with overlaps between the ranges between the ranks.

**Table 4.** Results of bootstrapping

Bacterial pair	Proportion	2.5%**CI**	Mean	Median	97.5%**CI**
*E.coli*, *K.pneumoniae*	24.324	17.117	24.431	24.324	32.432
*E.cloacae*, *E.coli*	17.117	9.91	17.065	17.117	24.324
*E.cloacae*, *K.pneumoniae*	12.613	6.306	12.692	12.613	18.919
*E.coli*, *K.oxytoca*	7.207	2.703	7.123	7.207	11.712
*E.cloacae*, *K.oxytoca*	5.405	1.802	5.255	5.405	9.054

Credible range of the top 5five most common bacterial host pairings in the within-–patient population where SNPs=0.

**Fig. 9.**
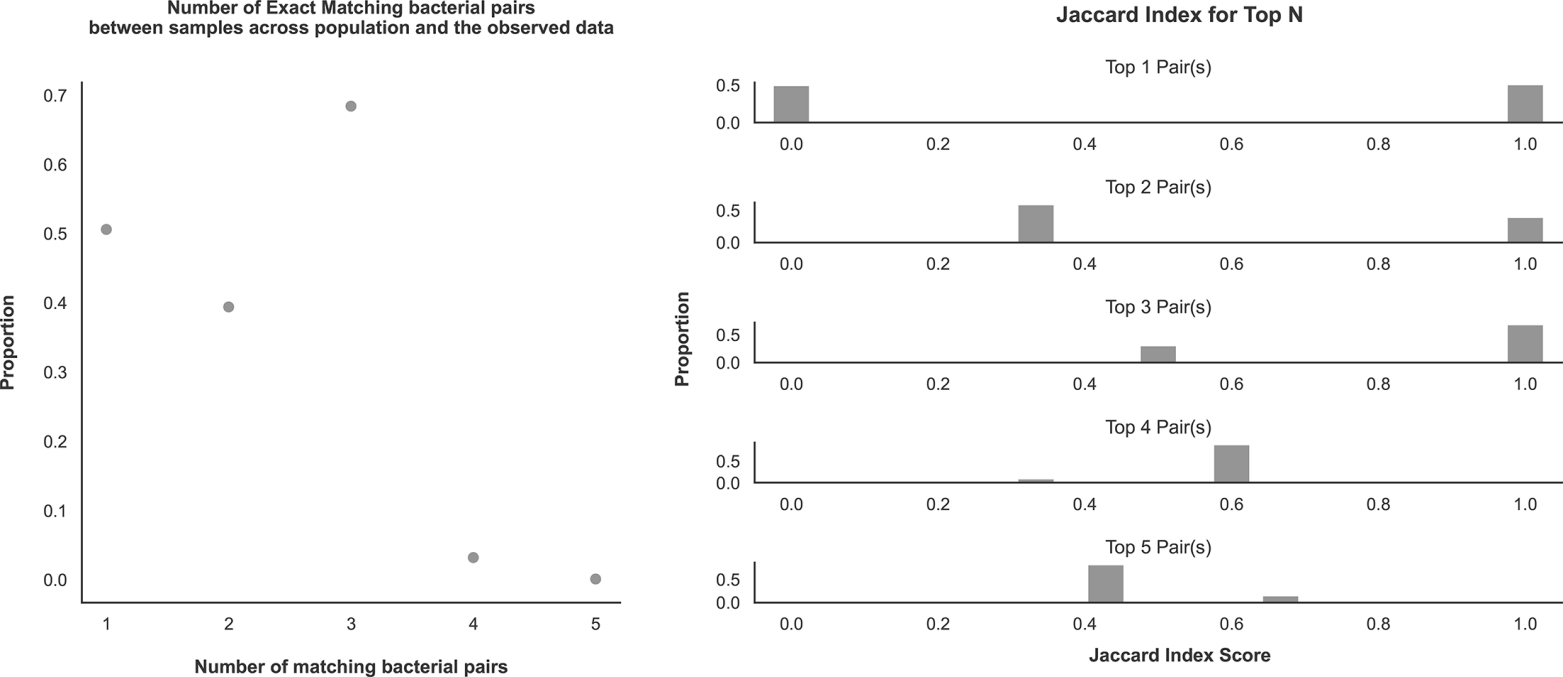
Jaccard similarity index scores. Plots showing the Jaccard similarity index after bootstrapping exact matching bacterial host pairs within the same patient across the population and the observed data. The scores shown are for the top 1 to 5 bacterial pairs.

Sampling across the whole population of bacterial host pairs where SNPs=0 shows that the top three observed pairs within the observed sample are more likely to appear. This is represented by both the exact match results and the range of the Jaccard index, as shown in Fig. 9 – a median index score of 1 may indicate that the top three bacterial pairs occur over half of the time. This could suggest that the following bacterial pairs – ‘*E. coli* and *K. pneumoniae*’, *‘E. cloacae* and *E. coli*’ and ‘*E. cloacae* and *K. pneumoniae*’ – are more likely to pose a higher risk of plasmid conjugation. However, these top three bacterial host pairs we observe are simply the combinations of the most abundant bacterial hosts in the dataset. Due to the limitations of the size of the dataset and potential over-representation of these pairings, we cannot determine with any significant statistical certainty if any combination of bacterial hosts is more likely to share the plasmid by conjugation.

### Molecular analysis

**Fig. 10.**
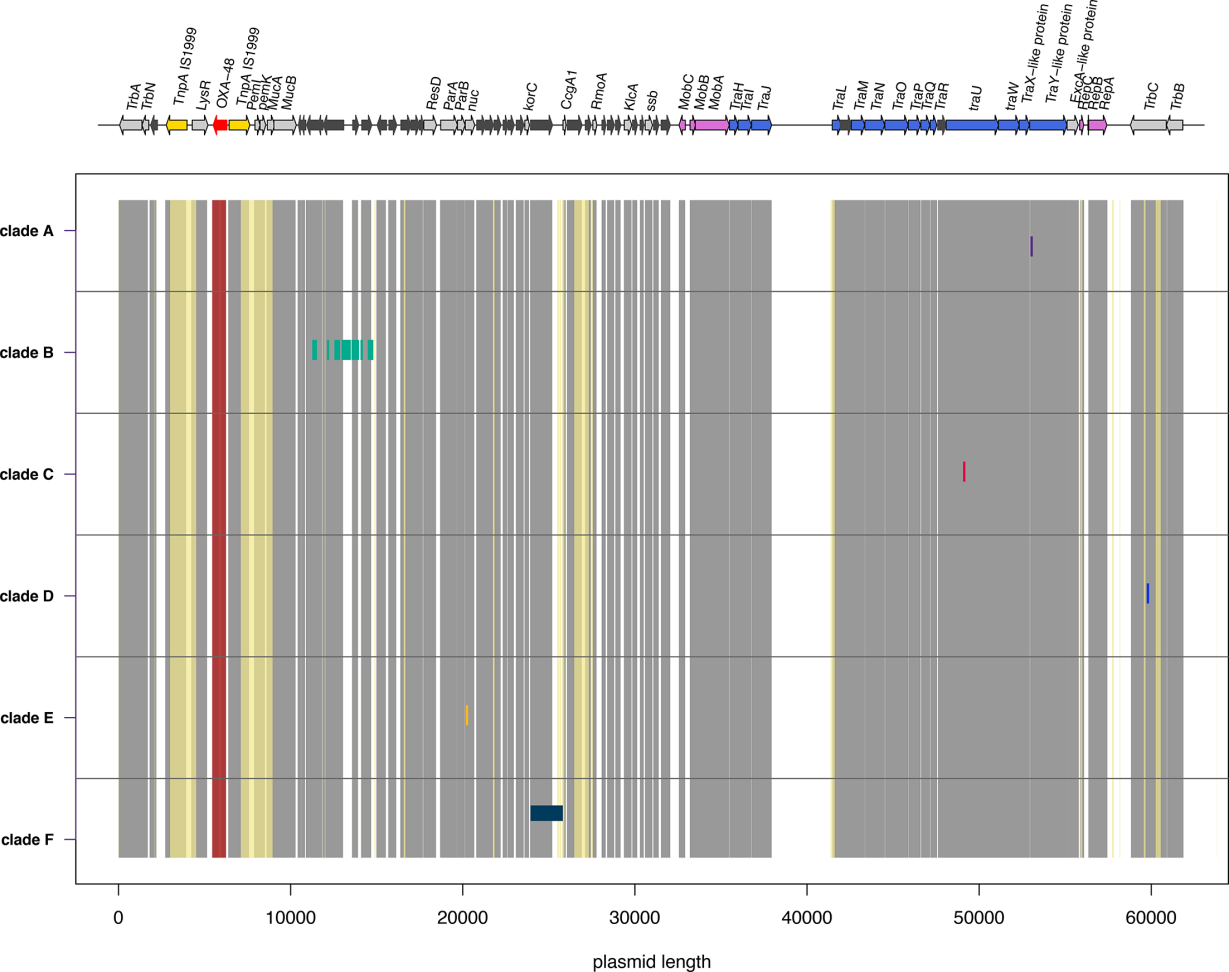
Mutations characterizing subclades. Mutations characterizing the subclades in Fig. 5. The annotated plasmid is shown at the very top of the figure. In dark grey, we coloured the genes annotated as hypothetical, in red the OXA-48 resistance gene, in yellow the TnpA IS1999 gene, in blue all the Tra genes, in pink all the Mob genes and in light grey all the remaining annotated genes. The subclades have the same labels (**A**–F) and colours as in Fig. 5. The grey vertical bars indicate all the annotated genes in the plasmid. The yellow vertical bars indicate that the region has been ‘masked’ (see File S1). The region of the OXA-48 gene present in the plasmid sequence is shown with the red vertical bar. Subclade-specific mutations are highlighted with short vertical bars in the same colour as the subclade highlights in Fig. 5.

To try to understand if adaptation was driving the main subclades in the epidemic clade (labelled A–F in Fig. 5), the mutations characterizing them were analysed. These mutations are shown in Fig. 10. Four of the six subclades are characterized by just one nucleotide substitution (A, C, D and E). The substitution characterizing subclade A is located in the coding region of a protein annotated as TraY-like. Substitutions characterizing subclades C, D and E are located in coding regions of the following genes: TraU, TrbC and nuc. At least two of these mutations are in genes that have been described to acquire mutations in previous studies: mutations in the gene nuc have been described in Ledda *et al*. [15] and mutations in TraU and TraY have been described in DelaFuente *et al*. [10]. Subclade B is characterized by 34 mutations in a 3500 bp region (from base 11 356 to base 14 754) where hypothetical genes have been annotated by León-Sampedro *et al*. [44], most probably due to homologous recombination. Subclade F is characterized by a 1567 bp long deletion in a hypothetical gene and a non-coding region (from base 24 017 to base 25 785). This deletion is overlapping with a hypervariable region in the same plasmid described in previous studies [15]. The fact that some of the mutations found in this study are in genes or intergenic regions already described as hosting mutations in other studies suggests that those regions are more prone to mutations and that these mutations might be adaptive.

## Discussion

In this article, we have analysed a within–patient dataset of the pOXA-48 plasmid, finding that conjugation of identical plasmids in different bacterial hosts has a strong role in pOXA-48 dissemination. Reconstructing and comparing the plasmid in different sequences, we aimed at shedding light on the within–patient dynamics and evolution of this very well-conserved plasmid. We found that 85 of the 115 patients in our dataset carried at least two identical copies of the plasmid in different bacterial hosts. Only 2 of the 115 analysed patients had variants of the plasmid belonging to different clades of the tree, one from the ‘diverse’ clade and one from the ‘epidemic’ clade. Such a diversity in the plasmid sequence can only be explained by multiple infections.

Similar results on pervasive within–patient conjugation were obtained by León-Sampedro *et al*. [44] on a large dataset of within-hospital periodical screening. While the analysis cannot be directly compared as their dataset offered leeway for much more detailed analysis, their study also finds widespread within–patient conjugation. It is interesting to note how the most abundant *Klebsiella* and *E. coli* strains are found in both datasets but with different abundances, which might point to the plasmid getting adapted to different strains at different geographical locations (2014–2016 in Madrid León-Sampedro *et al*. [44] and 2014–2016 in England this study). Also interesting to note is that they do not find any conserved structural variation that could be used to study within–patient transmission, while we find that one of the six subclades in our outbreak clade (subclade F is characterized by a 1567 bp long deletion). Interestingly, the two reference plasmids in this study and in León-Sampedro *et al.*’s study have the insertion sequence containing the resistance gene inserted in opposite directions (see Fig. S1). This is a feature that would need further investigation, far beyond the scope of this article. In general, a blast search for our epidemic plasmid did not find any identical plasmids. A tree of the closest 250 plasmids (more than 95% coverage and 99% identity) found in said search is shown in Fig. S2. This is an interesting result, pointing to the fact that our epidemic clade is spreading in England. The closest plasmids found in this search had been sampled in different European locations (Switzerland [58] and the Netherlands [59] mainly) and none in England.

The fact that the vast majority of patients have the same plasmid in different bacterial hosts has strong implications for public health investigations of pOXA-48 outbreaks: we find that in most patients the same allele of the plasmid is carried in different bacterial hosts. That means that, if we had sequenced just one sequence per patient, by solely focusing on the plasmid, we would have been able to identify at least 85/107 (79%) patients we found in the outbreak clade and which ones were carrying plasmids from the ‘diverse’ clade. On the other hand, if we had focused on sampling just resistant sequences from a specific bacterial host, like *E. coli* or *K. pneumoniae*, we would have gotten a much more reduced vision of the outbreak, identifying 61 and 56 patients, respectively, in the epidemic clade. Other studies show that this is not true for other plasmids [60], which display a wider within–patient genomic variability. A better understanding of the within–patient plasmid variability and the role of transmission bottlenecks is clearly of paramount importance to develop effective investigation protocols for plasmid-mediated AMR outbreaks.

Of course, the dataset has some limitations: multiple sequences per patient were sequenced either if they carried different carbapenemase genes in the same bacterial host or if they carried the same carbapenemase gene in a different bacterial host [1]. A very important part of the dynamics is therefore missing: how many times the same plasmid variant would have been identified in the same bacterial host had more than one colony pick per bacterial isolate been sequenced per patient. While knowing this would have changed the percentages in our results, it would not have changed the most important result of this analysis: that a patient very rarely acquires two different alleles of the pOXA-48 plasmid. We are, of course, not able to determine whether we find the same plasmid in two different bacterial hosts because the conjugation happened in the sampled patient or because the sampled patient was passed both hosts during the infection and the conjugation happened in another patient. This is, in a sense, not relevant, as the main point to take home is that this plasmid is frequently conjugating and the odds of finding it in very different hosts are quite high, and this is what is important for devising appropriate screening protocols. Research on plasmid conjugation is at a turning point. One of the main problems so far is that many of the *in vitro* results cannot be even compared among themselves due to the different units in which they are expressed [20,21]. New methods to study *in vitro* conjugation are being developed [20,21], which will lead to a meaningful comparison of the conjugation rate of different plasmids. Although in this case, we haven’t been able to compute a rate for the within–patient conjugation, we showed that it is widespread in our dataset. The limited variability of pOXA-48 has offered a unique possibility of avoiding all the sequencing and reconstruction issues affecting other plasmids to catch a glimpse of its within–patient dynamics [10]. With long read sequencing techniques becoming more and more reliable, such studies will be available for other plasmids and we will gain a clearer picture of within–patient plasmid dynamics.

This study shows how important it is to have a thorough understanding of the role of conjugation in plasmid-mediated AMR outbreaks [61]. It might inform sampling strategies in an outbreak investigation that can lead to a better understanding of the outbreak dynamics. By clarifying where the resistance is harboured and how often the plasmid conjugates, we could gain a better understanding of the effectiveness of potential approaches to mitigate an outbreak. Plasmid conjugation is the reason why plasmid-mediated AMR outbreaks are so concerning from the public health perspective. The more we understand about within–patient conjugation, the better tools we can develop for effective investigation of such outbreaks.

## supplementary material

10.1099/mgen.0.001361 Uncited Fig. S1.

## Abbreviations

AMR: antimicrobial resistance
CPE: carbapenemase-producing *Enterobacterales*
GTR: generalized time reversible
HGT: horizontal gene transfer
OXA-48: Oxacillinase variant 48 (the gene of interest, conferring carbapenem resistance) pOXA-48 plasmid carrying the OXA-48 gene of interest
ST: sequence type
